# W-Band Photonic Receiver for Compact Cloud Radars

**DOI:** 10.3390/s22030804

**Published:** 2022-01-21

**Authors:** Dmitry Strekalov, Ninoslav Majurec, Andrey Matsko, Vladimir Ilchenko, Simone Tanelli, Razi Ahmed

**Affiliations:** Jet Propulsion Laboratory, California Institute of Technology, MS 298-100, Pasadena, CA 91109, USA; Ninoslav.Majurec@jpl.nasa.gov (N.M.); andrey.b.matsko@jpl.nasa.gov (A.M.); vladimir.s.iltchenko@jpl.nasa.gov (V.I.); simone.tanelli@jpl.nasa.gov (S.T.); razi.u.ahmed@jpl.nasa.gov (R.A.)

**Keywords:** RF-photonic receiver, whispering gallery resonator, microwave up-conversion, cloud remote sensing

## Abstract

We introduce an RF-photonics receiver concept enabling the next generation of ultra-compact millimeter wave radars suitable for cloud and precipitation profiling, planetary boundary layer observations, altimetry and surface scattering measurements. The RF-photonics receiver architecture offers some compelling advantages over traditional electronic implementations, including a reduced number of components and interfaces, leading to reduced size, weight and power (SWaP), as well as lower system noise, leading to improved sensitivity. Low instrument SWaP with increased sensitivity makes this approach particularly attractive for compact space-borne radars. We study the photonic receiver front-end both analytically and numerically and predict the feasibility of the greater than unity photonic gain and lower than ambient effective noise temperature of the device. The receiver design is optimized for W-band (94 GHz) radars, which are generally assessed to be the primary means for observing clouds in the free troposphere as well as planetary boundary layer from space.

## 1. Introduction

Climate and weather models depend on high resolution (ideally, an order of hundreds of meters) and frequent (ideally, an order of minutes) spaceborne satellite measurements of clouds and precipitation. Such observations are necessary for use in operational forecasts and for validation and improvement of the fundamental equations that describe the models themselves. Trailblazing missions such as the Tropical Rainfall Measuring Mission (TRMM) [1], CloudSat [2,3] and Global Precipitation Measurement (GPM) [4] have demonstrated the central role of cloud and precipitation radars in this context. Groundbreaking improvements in weather and climate models have resulted from the data collected by these instruments.

This first generation of spaceborne cloud and precipitation radar systems had a limitation related to their size weight and power (SWaP). These instruments were implemented only in single units and were unable to cover the rapid temporal evolution of weather systems from low Earth orbit. So far, that function has been limited to lower resolution passive microwave and visible/infra-red sensors. Recent studies pertaining to cloud science and its global observation from space are focusing their attention on compact W-band radar systems [5] as key elements of their complement of instruments, as they can be implemented and deployed with either reduced cost or in larger numbers. In general, there is an unmet need for high performance, compact, W-band radars to observe clouds at sufficient spatial (vertical and horizontal) and temporal resolution.

In this paper, we provide the architectural and analytical framework for the development of microwave-photonic frequency converters, which are identified as instrumental for the development of compact millimeter wave radars that fill this unmet need. The converter discussed here involves an optical resonator interacting with the W-band signal of interest, as well as with a monochromatic optical carrier. The microwave signal is upconverted to the optical frequency domain and is processed optically [6,7,8,9,10,11,12,13,14,15,16,17,18,19,20,21,22]. Since the optical parts have small sizes and are characterized by low attenuation and negligible thermal noise, the entire photonic system has much smaller dimensions, as well as superior performance, compared to a pure electronic implementation.

The photonic device becomes feasible due to the availability of high quality (Q-) factor micro-resonators made with electro-optic materials. For instance, optical whispering gallery mode (WGM) resonators made out of LiNbO${}_{3}$ and LiTaO${}_{3}$ have been successfully used in prototypes of functional devices for optical and microwave photonic applications. Millimeter-sized WGM resonators are characterized by optical bandwidth in the hundred kilohertz (weakly coupled) to gigahertz (fully loaded) range. The outstanding optical transparency as well as the high electro-optical non-linearity of lithium tantalate and niobate enables the realization of a number of high-performance photonic microwave receivers [6,7,23,24,25,26,27,28,29,30,31]. The resonant interaction of a few optical WGMs with a microwave or millimeter-wave signal was supported by optimally shaping a microwave resonator coupled to an optical WGM resonator.

Both microwave and optical resonators are utilized to increase the efficiency of the electro-optical interaction occurring within limited frequency bands around the respective microwave and optical carrier frequencies [32,33,34,35,36,37,38,39]. Importantly, the efficiency of the interaction increases with the size reduction of the components. Ultimately, the photonics components should become quasi-lumped elements. Electro-optically active WGM resonators are attractive for this application because they feature high quality factors at any optical frequency within the transparency window of the resonator host material while having a size comparable or less than the RF wavelength [6,23,24,25,26,27,28,29,30,31,40,41,42,43,44,45]. These devices operate either at the optical baseband (see, e.g., [43]) or at bands detuned from the baseband by the frequency difference between modes of the WGM resonator. The latter versions of the all-resonant electro-optic frequency converters have the highest efficiency at higher microwave frequencies (see, e.g., [16,20,25,29,45]).

Upconversion of the microwave signal to the optical domain in all-resonant electro-optical devices occurs because the microwave field selectively interacts with modes of a nonlinear optical WGM resonator. The efficiency of the transformation is proportional to $Q^{2}Q_{M}$ [6], where *Q* and $Q_{M}$ are the loaded quality factors of the optical and microwave modes, respectively. Therefore, the higher the quality factors are, the smaller the microwave power has to be for achieving the same modulation efficiency, or in other words, the higher the microwave-to-optics conversion efficiency will be.

RF photonic receivers are promising for practical applications because their performance fundamentally does not depend on the RF frequency. Nonlinear WGM resonators have been used in photonic front-end receiver applications operating in the frequency bands ranging from X- to Ka- and W-bands. Theoretically, the lithium niobate and tantalate WGM-based mixers operate well at RF frequencies ranging from several GHz to 1 THz. The high frequency limitation is defined by the THz transparency range of the material.

To date, the noise temperature of the demonstrated photonic receivers has far exceeded ambient temperature. The performance was limited because of the non-optimal design of the photonic structures. We have found that an optimal design of the receiver can allow for a noise temperature below the ambient 300 K. The form factor of the physics package of the device can be as small as 3 cc, and the power consumption can be below 1 W. We envision that a 94 GHz cloud radar with such a receiver can have a system noise temperature below 300 K, assuming that the radar itself adopts a very low loss front end architecture, as shown in the next section. Such low noise temperatures can result in approximately 4 dB better sensitivity than typical W-band radar implementations while also reducing instrument SWaP.

The paper is organized as follows. In Section 2, we describe the major operation principles of the resonant W-band photonic receiver based on a WGM resonator. In Section 3, we present a theoretical analysis of the receiver’s performance. Section 4 is devoted to theoretical calculations of the device sensitivity. The numerical simulations of a particular receiver configuration are described in Section 5. Section 6 concludes the paper.

## 2. Concept of Operation

**Figure 1:**
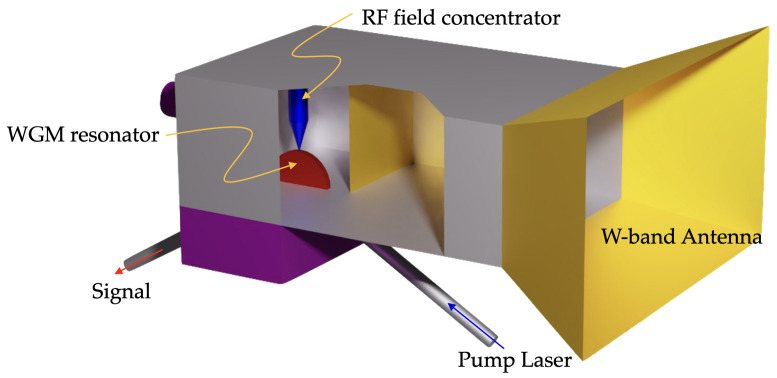
Conceptual design of the W-band microwave photonic receiver. A WGM resonator is interrogated by coherent light. A signal of interest enters the horn antenna and propagates to the WGM resonator, reaching high intensity in the vicinity of the resonator surface due to a special configuration of RF field concentrator electrodes. Due to the electro-optical effect, the signal is upconverted to the optical frequency domain and leaves the resonator through the optical port. The signal is subsequently optically processed and downconverted to the IF domain (not shown in the diagram).

The receiver architecture is illustrated by Figure 1. The signal of interest is collected by a directional horn antenna. The output of the antenna is tapered down and coupled to a W-band hollow waveguide. The waveguide is terminated with a resonant section that contains an electro-optical transducer that upconverts the W-band signal to optical frequencies. The transducer consists of a WGM resonator fabricated out of the electro-optical material. A mode of the resonator is interrogated optically via the pump laser. The electric field of the input RF signal generates an optical harmonic detuned from the optical carrier by the frequency of the RF signal matching the frequency of another optical WGM. The process is greatly enhanced when the geometrical distributions of the RF and optical fields properly overlap. In what follows, we show that a configuration that involves transverse electric (TE) and transverse magnetic (TM) WGMs is an advantageous one, as the frequency difference between the modes can be tuned with temperature or DC voltage applied to the resonator. Further enhancement of the interaction can be achieved by the optimal shaping of the electrode integrated with the waveguide. The optical harmonic generated in the nonlinear WGM resonator propagates with the light leaving the resonator and is converted to an intermediate frequency (IF) by mixing the light with a local oscillator (LO) field on a fast photodiode. We estimate the sensitivity of this type of a measurement in subsequent sections.

## 3. Theoretical Performance Model

Our approach is based on using orthogonaly polarized optical pump and signal WGMs: one from the TE family, the other from the TM family. In a resonator fabricated from a birefringent optical crystal (such as lithium niobate or lithium tantalate) so that the optical axis is also the resonator axis of symmetry, the TE-polarized light “sees” predominantly the extraordinary index of refraction $n_{e}$ while the TM-polarized light “sees” predominantly the ordinary index of refraction $n_{o}$. These two indices have different temperature dependencies and also different dependencies on the external DC electric field. Therefore, manipulating these two control parameters, we can tune the TE and TM WGMs frequencies relative to each other, achieving the desired frequency detuning.

To build the theoretical model, we start by deriving the electro-optical interaction Hamiltonian, which will yield the coupled WGMs equations. The interaction energy of the optical TE and TM WGMs fields ${\vec{E}}_{TE}$ and ${\vec{E}}_{TM}$ and the electric field of the RF signal under study ${\vec{E}}_{RF}$ can be presented as the following volume integral [46]:

(1) $$\begin{array}{c} E=\frac{n_{e}^{2}n_{o}^{2}}{4\pi }\int_{V}r_{51}\left({\vec{E}}_{TM}\cdot {\vec{E}}_{RF}\right)E_{TE}\;dV, \end{array}$$

where ${\vec{E}}_{TE}=\hat{z}E_{TE}$ and ${\vec{E}}_{TM}=\hat{\rho }E_{TM}$, $\hat{z}$ and $\hat{\rho }$ being the unit vector in the optical axis direction and perpendicular to its radial direction. The interaction mediated by the electro-optic tensor elements $r_{42}=r_{51}$ requires the RF field to have a radial component. For the sake of simplicity, we assume that ${\vec{E}}_{RF}=\hat{\rho }E_{RF}$. It is also convenient to use esu units for optical fields and SI units for $r_{51}$ and $E_{RF}$. Note that the space averaged interaction energy E can zero down because of the phase mismatch between the light and the signal RF field.

Each electric field in (1) has both positive and negative frequency components. If a field corresponds to an excitation of an optical mode, its amplitude can be quantized, that is, represented through the photon’s creation and annihilation operators $a^{†}$ and *a* and the eigenfunction of this mode Ψ:

(2) $$E_{TE}\left(\vec{r}\right)=\frac{\sqrt{2\pi \hbar \omega_{TE}}}{n_{e}}\left(e^{i\omega_{TE}t}\Psi_{TE}\left(\vec{r}\right)a_{TE}^{†}+e^{-i\omega_{TE}t}\Psi_{TE}^{*}\left(\vec{r}\right)a_{TE}\right),$$

and similarly for $E_{TM}\left(\vec{r}\right)$. The TE and TM eigenfunctions $\Psi_{TE,TM}$ are normalized to unity:

(3) $$\int_{V}{\left|\Psi {\left(\vec{r}\right)}_{TE,TM}\right|}^{2}\;dV=1,$$

and therefore, have the units of cm${}^{-3/2}$.

While the optical WGM eigenfunctions are very accurately approximated by the well-known analytical expressions introduced below, the microwave mode eigenfunctions are not readily available in our geometry. Instead, we will use the electric field distribution $E_{RF}\left(\vec{r}\right)$ found from the simulations using Ansys High Frequency Structure Solver (HFSS). Only the radial projection of this field should be taken into account.

Adopting the rotated-wave picture, we can write the interaction Hamiltonian for single side-band modulation as follows:

(4) $${\hat{H}}_{int}=\hbar g\;{\hat{a}}_{TE}{\hat{a}}_{TM}^{†}+h.c.,$$

where the coupling constant *g* is:

(5) $$\begin{array}{cc} g & =n_{o}n_{e}r_{51}\sqrt{\omega_{TE}\omega_{TM}}\int_{V}\Psi_{TE}^{*}\left(\vec{r}\right)\Psi_{TM}\left(\vec{r}\right)E_{RF}\left(\vec{r}\right)\;dV \end{array}$$

(6) $$\begin{array}{cc} \; & \approx n_{o}n_{e}r_{51}\frac{2\pi c}{\lambda }\int_{V}\Psi_{TE}^{*}\left(\vec{r}\right)\Psi_{TM}\left(\vec{r}\right)E_{RF}\left(\vec{r}\right)\;dV, \end{array}$$

where $\lambda_{TE}\approx \lambda_{TM}=\lambda$ is the optical wavelength in vacuum. The integral is taken over the resonator volume, where the effective electro-optical constant $r_{51}$ is not zero.

The analytical approximations for the optical WGM eigenfunctions are available from [47] in the form:

(7) $$\Psi \left(\chi ,\xi ,\phi \right)\propto e^{im\phi }e^{-\xi^{2}/2}H_{L-m}\left(\xi \right)\mathrm{Ai}\left(\chi -\alpha_{q}\right),$$

where $H_{L-m}\left(\xi \right)$ is a Herimitian polynomial, Ai(χ) is the Airy function which has positive-valued zeros $\alpha_{q}$, i.e., $\mathrm{Ai}(-\alpha_{q})=0$ for q=1,2,…. The scaled coordinates χ and ξ are defined as:

(8) $$\chi \approx 2^{1/3}m^{2/3}\frac{w}{R},\;\xi =\sqrt{m}{\left(\frac{R}{\rho }\right)}^{1/4}\theta ,$$

where *R* and ρ are the resonator radius and the local curvature of its rim, respectively. The angle θ is measured along the rim curvature starting from the equator, and the coordinate *w* is measured from the resonator surface towards the center of the rim curvature. The WGM numbers are the L,m,q. For the best conversion efficiency, we need to find such WGMs that L=m (which makes $H_{L-m}\left(\zeta \right)=1$) and q=1, both for the optical pump and signal. The remaining mode number can be estimated from the resonator size and the optical wavelength: m≈2πnR/λ, so the scaled coordinates (8) can be written in the following form:

(9) $$\chi \approx 2{\left(\frac{\pi n}{\lambda }\right)}^{2/3}R^{-1/3}w,\;\xi =\sqrt{2\pi n\frac{R}{\lambda }}{\left(\frac{R}{\rho }\right)}^{1/4}\theta .$$

A volume element in these coordinates can be written as dV≈Rρdwdθdϕ [47]. Then, (6) transforms to:

(10) $$g\approx n_{o}n_{e}r_{51}\frac{2\pi c}{\lambda }\frac{\int e^{i\Delta m\phi }e^{-\xi^{2}}{\mathrm{Ai}}^{2}\left(\chi -\alpha_{1}\right)E_{RF}\left(w,\theta ,\phi \right)dw\;d\theta \;d\phi }{2\pi \int e^{-\xi^{2}}{\mathrm{Ai}}^{2}\left(\chi -\alpha_{1}\right)dw\;d\theta },$$

where we neglected any difference in the pump and signal mode parameters, such as the refraction indices, wavelengths and mode numbers, *except* in the $e^{im\phi }$ term. This term yields the $e^{i\Delta m\phi }$ factor in the overlap integral in the numerator, with $\Delta m=m_{TE}-m_{TM}$, and points at the necessity to optimize the “interaction length” along the equator, which is determined by the width of the RF electrode.

To further simplify the analysis, we neglect the variation of the W-band field distribution $E_{RF}\left(w,\theta ,\phi \right)$ across the (θ,w) cross section of the optical mode, replacing $E_{RF}\left(w,\theta ,\phi \right)\to E_{RF}\left(w_{0},0,\phi \right)$, where $w_{0}$ is the depth of the Airy function maximum:

(11) $$w_{0}\approx 0.308{\left(\frac{R\lambda^{2}}{n^{2}}\right)}^{1/3}.$$

Then, the integrals in the numerator and denominator of (10) partially cancel, leaving us with:

(12) $$g\approx n_{o}n_{e}r_{51}\frac{c}{\lambda }\int_{-\pi }^{\pi }\cos\left(\Delta m\phi \right)E_{RF}\left(w_{0},0,\phi \right)d\phi .$$

Here, we assumed that the W-band field is symmetric with respect to ϕ=0 and θ=0 (that is, ϕ=0,θ=0 is a direction to the center of the W-band electrode).

As we mentioned earlier, the radial component of the W-band electric field $E_{RF}\left(w_{0},0,\phi \right)$ is found from the HFSS simulations and given to us directly in the units of V/m. This field is found based on the known W-band power $P_{W}$ injected into the waveguide. This power will serve as the reference level in determining the conversion efficiency. It is convenient to write the W-band field as $E_{RF}\left(w_{0},0,\phi \right)=E_{0}F_{RF}\left(w_{0},0,\phi \right)$, where $E_{0}=E_{RF}\left(w_{0},0,0\right)$ is the peak value of the RF field. This value corresponds to the W-band signal power $P_{RF\;in}=\alpha_{W}E_{0}^{2}$ through an empirical coefficient $\alpha_{W}$, which we find from the HFSS simulation. The function $F_{RF}\left(w_{0},0,\phi \right)$ is introduced such that $F_{RF}\left(w_{0},0,0\right)=1$ and is independent of $P_{RF\;in}$. It describes the W-band field’s geometrical profile and can be used for optimization of the RF electrode width.

The conversion rate *g* is the key parameter determining our system’s performance. To find the optical conversion efficiency, we write the slow-amplitude Langevin equations for the Hamiltonian (4):

(13) $$\begin{array}{cc} \dot{A} & =-\left(i\Delta \omega_{a}+\gamma_{a}\right)A-ig^{*}B+\sqrt{2\gamma_{ac}\frac{P_{0}}{\hbar \omega_{a}}},\\ \dot{B} & =-(i\Delta \omega_{b}+\gamma_{b})B-igA, \end{array}$$

where $A=\langle \hat{a}\rangle e^{i\omega_{a}t}$ and $B=\langle \hat{b}\rangle e^{i\omega_{b}t}$ are the slow amplitudes of the pump and signal optical fields, respectively; $\Delta \omega_{a,b}$ are the detunings of the pump and signal fields from their respective WGM resonances (we will assume that these are zeros); $\gamma_{a,b}$ are total loss rates for the optical modes; $\gamma_{ac}$ is the coupling rate for the pump mode; and $P_{0}$ is the external optical pump power injected into this mode. In general, depending on which polarization the pump has, *a* may correspond to TE and *b* TM or vice versa.

A stationary solution of the Langevin equations, (13), for the signal is:

(14) $$B=\frac{ig}{{\left|g\right|}^{2}-\gamma_{a}\gamma_{b}}\sqrt{2\gamma_{ac}\frac{P_{0}}{\hbar \omega_{a}}}\approx \frac{-ig}{\gamma_{a}\gamma_{b}}\sqrt{2\gamma_{ac}\frac{P_{0}}{\hbar \omega_{a}}}.$$

The approximation is made under the assumption that the coupling rate *g* is much smaller than the loss rate, which is expected to be the case. Note that the circulating signal amplitude becomes infinite as ${\left|g\right|}^{2}$ approaches $\gamma_{a}\gamma_{b}$. This arises from the the main drawback of our model, which is a lack of the back action of the optical field on the W-band field, or in other words $E_{RF}$ is not being affected by the optical pump. Howeverm, for low conversion efficiency, this is a good approximation, and our model is viable.

The output signal power $P_{s}$ is related to the circulating signal field as $P_{s}=2\gamma_{bc}\hbar \omega_{b}{\left|B\right|}^{2}$, so the optical sideband contrast is:

(15) $$\eta_{s}=\frac{P_{s}}{P_{0}}\approx \gamma_{ac}\gamma_{bc}{\left(\frac{2g}{\gamma_{a}\gamma_{b}}\right)}^{2}.$$

Considering that $\gamma_{a}=\gamma_{a0}+\gamma_{ac}$, and similarly for $\gamma_{b}$, it is easy to see that the maximum efficiency is reached for strongly over-coupled optical pumps and signals when $\gamma_{a}\approx \gamma_{ac}$ and $\gamma_{b}\approx \gamma_{bc}$. In this case:

(16) $$\eta_{s}=\frac{4g^{2}}{\gamma_{a}\gamma_{b}}.$$

To perform the measurement, we mix the optical modulation sideband with a local oscillator field on a balanced fast photodiode and measure the power of the beat note RF signal. This power is found as:

(17) $$\begin{array}{c} P_{RF\;out}=\rho R^{2}P_{0}P_{LO}\eta_{s}=\rho R^{2}P_{0}P_{LO}\frac{4g^{2}}{\gamma_{a}\gamma_{b}}, \end{array}$$

where R is the responsivity of the fast photodiode in A/W, and ρ is the resistance of the RF circuit interfacing the photodiode. It can be related to the W-band signal power $P_{RF\;in}$ supplied to the waveguide by introducing a photonic gain *G* as:

(18) $$G=\frac{P_{RF\;out}}{P_{RF\;in}}=\rho R^{2}P_{0}P_{LO}\frac{4g_{0}^{2}}{\gamma_{a}\gamma_{b}\alpha_{W}},$$

where we introduced the normalized coupling rate:

(19) $$g_{0}=\frac{g}{E_{0}}=n_{o}n_{e}r_{51}\frac{c}{\lambda }\int_{-\pi }^{\pi }\cos\left(\Delta m\phi \right)F_{RF}\left(w_{0},0,\phi \right)d\phi .$$

The chosen optical and RF electric fields orientations allow for two optical polarization options: the TE–TM conversion, when the optical pump is coupled into a TE mode of the resonator and the signal is generated in a TM mode, and the TM–TE conversion, when the pump is coupled into a TM mode and the signal is generated in a TE mode. Both options are equivalent in terms of the conversion efficiency, however usually $\gamma_{TE}>\gamma_{TM}$. This means that a higher circulating power can be afforded in the TE modes before the thermorefractive and photorefractive effects become too strong. Therefore, the TE modes are more suitable as the pump modes, and we will focus on the TE–TM type of conversion.

Both TE and TM modes’ eigenfrequencies are related to the mode numbers via the WGM dispersion equations [48]. These equations include the indices of refraction that themselves depend on the optical frequencies, which makes the equations transcendental. They have to be solved numerically. Our goal is to find such a pair of the TE (pump) and TM (signal) modes that the pump mode frequency matches the frequency of our laser and that the signal mode frequency is higher than the pump frequency by exactly the frequency of the expected RF signal ($f_{RF}=94.05$ GHz). This enables the anti-Stokes (up-converting) signal generation process, which is preferred over the Stokes (down-converting) process because the latter is prone to spontaneous generation of the optical signal even without the RF input.

This frequency-matching problem can be solved for discrete TE and TM WGM spectra thanks to the temperature tuning of the dispersion equations, achieved via the thermorefractivity. Our approach consisted of the following steps:

1. Find a pump (TE) mode nearest to the nominal pump wavelength λ=1558.6 nm at the nominal resonator temperature $T_{0}=35{\;}^{\circ }$C. This mode has a frequency $f_{p}$ which is no further from the nominal pump frequency than the resonator free spectral range (FSR). We assume that it can be accessed with a minimal tuning of the laser wavelength.
2. Find a signal (TM) mode nearest to the target frequency $f_{s}^{\left(0\right)}=f_{p}+f_{RF}$. Determine this mode’s frequency $f_{s}$.
3. Evaluate the RF frequency detuning of the found modes from the target RF signal frequency $\Delta f=f_{s}-f_{s}^{\left(0\right)}$. Keep the solution if the detuning falls into a specified frequency range which is deemed accessible.

**Figure 2:**
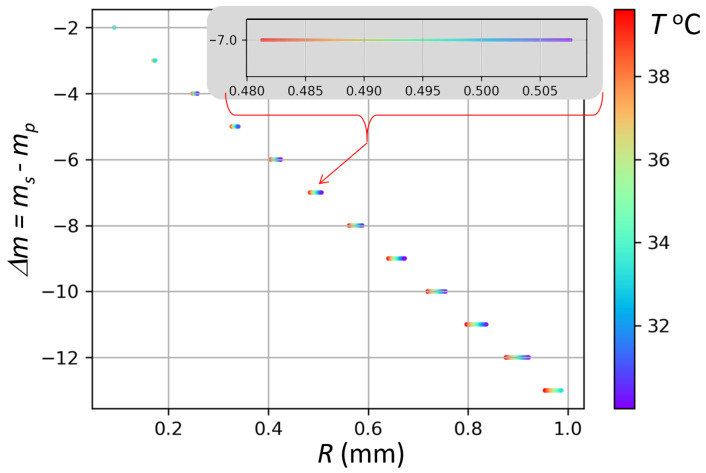
WGMs with different relative orbital numbers can be used for the 94.05 GHz RF signal upconversion in a TE–TM anti-Stokes process in Lithium Tantalate resonators of different radii at different temperatures.

The accessible RF frequency range is established based on the differential thermorefractivity $dn_{o}/dT-dn_{e}/dT$ and a reasonable range of the resonator temperature $T_{0}\;\pm \;\Delta T$. Assuming $T_{0}=35{\;}^{\circ }$C and $\Delta T=5{\;}^{\circ }$C, we arrive at the result shown in Figure 2. The temperature indicated by the color bar corresponds to achieving the desired microwave frequency $f_{s}-f_{p}=f_{RF}$ in a resonator of a given radius.

From Figure 2, we note that a larger resonator allows for a larger radius uncertainty for a fixed temperature range. In practice, limited fabrication precision of the resonators leads to a few microns uncertainty in the radius value. Therefore, a large radius tolerance is advantageous for resonator fabrication, although it implies the need for tighter temperature control in larger resonators.

The wavelength and temperature dependence of the indices of refraction varies with the crystal composition, which adds to the fabrication uncertainty. In this analysis, we use the Sellmeier equation for nominally pure Lithium Tantalate from [49]. If instead we used the Sellmeier equation for 1.0 mol% MgO-Doped stoichiometric Lithium Tantalate from [50], we would have found that the Δm=−2 frequency matching solution remains viable for the resonator radius ranging form 0.35 to 0.9 mm. That is the range sparsely populated by Δm=−12 to Δm=−5 solutions in Figure 2.

Fortunately, the large discrepancy which may be caused by the variation of the refractive properties among different samples does not significantly affect the performance of the receiver. It only affects our ability to find the desired pair of modes. This can be helped by the so far unused degree of freedom: the laser wavelength, which can be tuned much further than by a single FSR. We plan to use this free parameter to mitigate the fabrication and material-composition uncertainties.

## 4. Receiver Sensitivity

**Figure 3:**
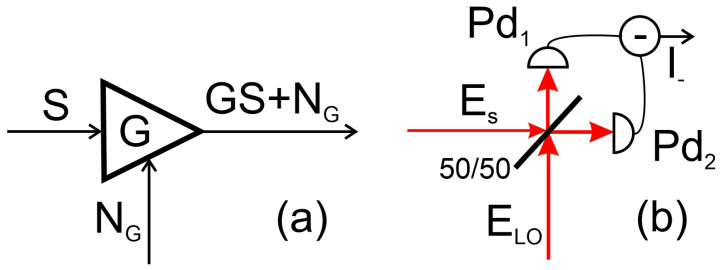
(**a**) Schematic of the receiver. The signal from the antenna goes through the photonic circuit that changes the magnitude of the signal and also adds noise associated with the temperature of the electronic circuit. (**b**) Schematic of the homodyne detection scheme involving a balanced photodiode.

To determine receiver sensitivity, we model the receiver and the additive noise sources in the form shown in Figure 3a. The signal (*S*) from the antenna is going to the photonic circuit that amplifies (or attenuates) it by the gain factor *G* given by (18). It also adds noise $N_{G}$ defined by the Johnson–Nyquist (thermal), as well as optical (shot and intensity) noise.

To evaluate the noise, let us consider the scheme shown in Figure 3b. The photo current in photodiodes $Pd_{1,2}$ can be written in the following form:

(20) $$\begin{array}{c} i_{1}=R{\left(E_{s}+E_{LO}\right)}^{2}/2, \end{array}$$

(21) $$\begin{array}{c} i_{2}=R{\left(E_{s}-E_{LO}\right)}^{2}/2, \end{array}$$

where R is the photodiode responsivity in A/W, $E_{s}=\sqrt{P_{s}}e^{i\phi_{s}}$ and $E_{LO}=\sqrt{P_{LO}}e^{i\phi_{LO}}$, $P_{s}$ and $P_{LO}$ are the power values for the signal and the local oscillator, respectively, and $\phi_{s}$ and $\phi_{LO}$ are their respective phases. The differential current is:

(22) $$\begin{array}{c} i_{-}=2RE_{LO}E_{s}. \end{array}$$

From this expression, we see that for the case of a weak signal, the noise of the local oscillator does not contribute to the noise of the differential current since the expectation value of the signal is small. At the output of the receiver, we have the following signal and noise powers:

(23) $$\begin{array}{ccc} & & S_{out}=4\rho R^{2}P_{LO}P_{s}=4\rho R^{2}P_{0}P_{LO}\frac{4g_{0}^{2}}{\gamma_{a}\gamma_{b}\alpha_{W}}P_{RF\;in}=4GP_{RF\;in}, \end{array}$$

(24) $$\begin{array}{ccc} & & N_{out}=N_{G}=\left[k_{B}T_{rec}+2\hbar \omega \rho R^{2}P_{LO}\right]\Delta F \end{array}$$

where ρ is the circuit impedance, ΔF is the reception bandwidth (which is always smaller than the bandwidth of the WGM coupled to the modulation sideband and the bandwidth of the RF resonator), while $T_{rec}$ is the receiver’s ambient temperature. Please note that compared with a single photodiode, see Equation (17), the balanced detection increases the signal by four times and the noise by two times.

The receiver sensitivity can be determined by setting the signal and noise powers as equal, i.e., $S_{out}=N_{out}$, leading to:

(25) $$\frac{P_{RF\;min}}{\Delta F}=\frac{k_{B}T_{rec}}{4G}+\frac{\gamma_{a}\gamma_{b}\alpha_{W}}{4g_{0}^{2}}\frac{\hbar \omega }{2P_{0}},$$

which suggests that in order to have a lower than ambient receiver noise temperature, the device must have a larger than unity gain for a receiver based on a single photodiode or G>0.25 for a balanced detector such as the one chosen in this receiver. Shot noise, likely the dominant noise source in this case, can be reduced, if needed, by increasing the pump power, $P_{0}$.

The actual minimum detectable signal, of course, depends on the receiver bandwidth, ΔF. Typical weather radars, for instance, have bandwidths on the order of a few MHz, and the RF-electronics-based receivers have noise figures ranging from 5 to 10 dB [51,52]. The typical minimum detectable power for a single pulse in a radar with 10 MHz bandwidth, a 6 dB noise figure and a receiver temperature of 300 K is approximately −97 dBm. For a photonic receiver such as the one being proposed here, the minimum detectable power for the same bandwidth, as will be shown in Section 5, can be as low as −110 dBm, an improvement of more than 10 dB. For compact radars, an increase in sensitivity of an order of magnitude while decreasing overall instrument size is very appealing.

Finally, let us point out that the receiver sensitivity analysis provided here is based on a conservative approach considering the full power of the electromagnetic fields thermal fluctuations within a specific frequency band as the noise. Alternatively, sometimes the mean-value of the additive noise can be “calibrated out”, providing a new zero level for the signal measurement. In this case, only the fluctuations of the noise power around its mean-value obscure the signal and should be compared against it. This approach, explored in [17,21], can potentially provide an even better sensitivity.

## 5. RF Simulations and Results

**Figure 4:**
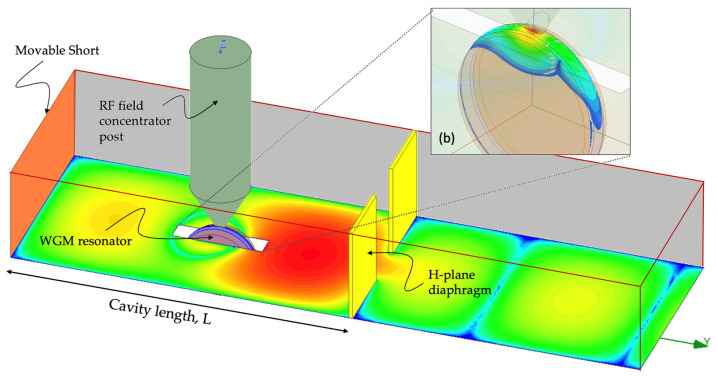
Structural model of the resonant section of the electro-optical transducer in HFSS with the WGM resonator and RF-field concentrator post. The wave-port excitation is to the right of the structure and the movable short is modeled as a PEC to the left. The H-plane diaphragm (also modeled as PEC plates) and the movable short create the waveguide cavity, parameterized by its length L. The strength of simulated E-field inside the waveguide structure is shown with the scaled colormap (red corresponding to the strongest field strength). Inset (b) shows the distribution of the simulated field inside the resonator.

To assess the performance of the electro-optical transducer a combination of detailed electromagnetic simulations using HFSS to estimate $E_{RF}\left(\vec{r}\right)$ at the W-band, and analytical models of optical WGM eigenfunctions, $\Psi_{TE,TM}\left(\vec{r}\right)$, are used in the theoretical model outlined in Section 3 and Section 4. The simulated resonant modulator structure is shown in Figure 4, with the RF concentrator post and the WGM resonator situated inside the standard WR-10 (2.54 mm × 1.27 mm) waveguide section with the waveguide walls treated as perfect electric conductors (PEC). A resonant waveguide cavity of length L is created inside the waveguide using a movable short (shown on the left of the structure in Figure 4) and an H-plane inductive diaphragm located to the right, both treated as PEC plates. The RF-field concentrator is a cylindrical metal post that tapers to the WGM resonator. The resonator, centered at the origin oriented so that its rim lies in the yz-plane, protrudes into the waveguide structure and is modeled as a dielectric structure with a tensor permittivity ${\tilde{\varepsilon }}_{r}$ matching that of LiTaO${}_{3}$ [53]. The HFSS simulated E-field strength inside the waveguide, based on an excitation port to the right in Figure 4 (not shown), is plotted as a scaled color-map, with red representing high and blue representing low field strengths. Of note is the relatively high field concentration inside the resonant cavity section. The distribution of the field inside the WGM resonator is shown in Figure 4 inset (b), with the RF energy concentrated in a small region around the tip of the field-concentrator post as desired.

**Figure 5:**
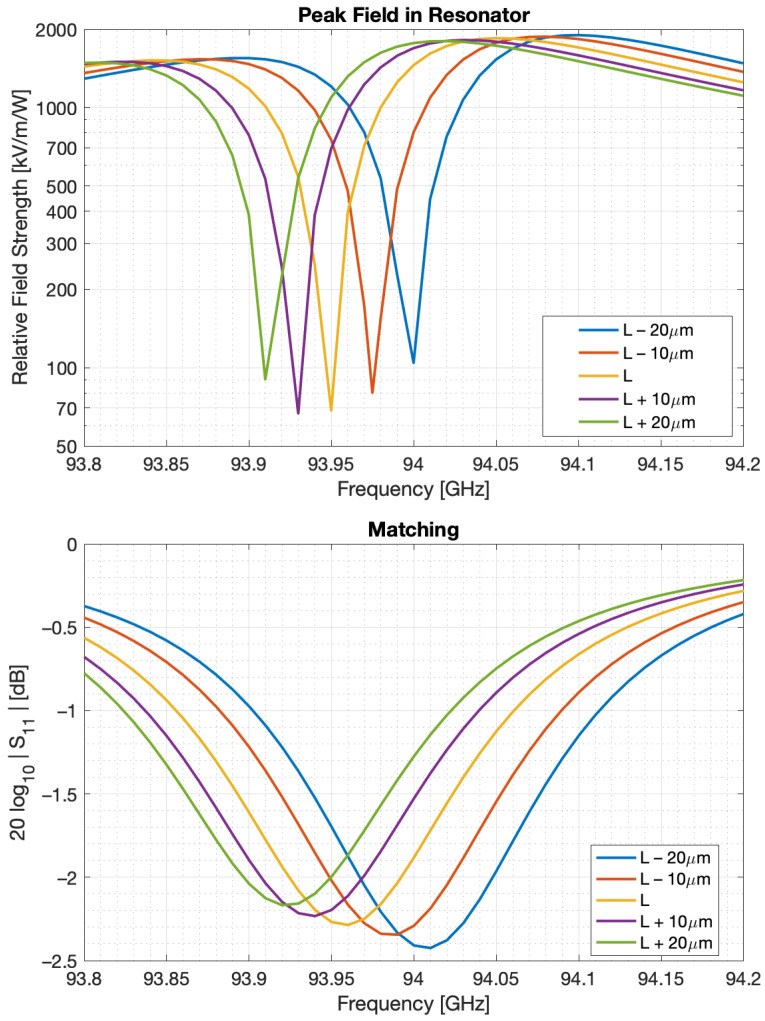
Simulated relative field strength approximately 2 μm inside the WGM receiver as a function of frequency for various positions of the matching stub. The field strength is described relative to the power (in Watts) at the input. The matching, $S_{11}$, of the resonant modulator is also shown.

Figure 5 shows the peak relative field strength, 2 μm inside the WGM resonator as a function of RF frequency centered around 94.05 GHz. Field strength is displayed in units of kV/m relative to an excitation power of 1W. The fields are plotted at a depth of 2 μm below the resonator rim since the optical radial window function peaks at that depth, thus most directly impacting the electro-optic coupling rate, *g* and the photonic gain *G*. Figure 5 also shows the sensitivity of the peak field to the length of the resonant cavity, L, which is equivalent to moving the shorting stub relative to its nominal position. This allows us the ability to tune the cavity length, L, and overcome fabrication tolerances. The nominal structure is designed to have a relative field strength peak of approximately 2000 kV/m/W at 94.05 GHz. In addition, shown in Figure 5 is the matching, or $S_{11}$, of the resonant structure. As evident from Section 3 and Section 4, matching itself does not directly impact receiver performance; however, observed variation in $S_{11}$ is useful as a stand-in for field strength, allowing us to tune the resonant structure using W-band measurements alone and not require optical measurement infrastructure during preliminary testing.

**Figure 6:**
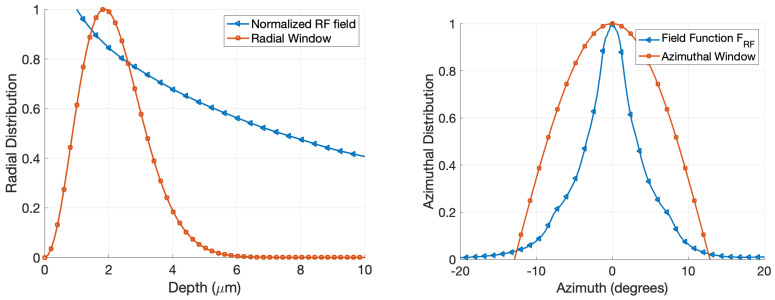
Simulated RF field strength and the radial window function vs. the depth (**left**). Simulated normalized RF field strength $F_{RF}\left(w_{0},0,\phi \right)$ evaluated at the optical WGM depth $w_{0}\approx 2\;\mu$m, and the azimuthal window function cos7ϕ (**right**).

Figure 6 shows the distribution of W-band fields inside the WGM resonator. As expected, these fields are concentrated around the tip of the post and dissipate outward. The left panel shows the radial distribution. Here, the depth *w* is measured from the resonator surface inwards in the equatorial plane of the resonator, see (7) and (8). The radial window function ${\mathrm{Ai}}^{2}\left(\chi -\alpha_{1}\right)$ is also shown. We see that the approximation made in the context of (10), which was to evaluate the RF field at the peak of the radial window function $w_{0}\approx 2\;\mu$m and then treat it as a constant, is sufficiently accurate. Similarly, the right panel shows the simulated azimuthal profile of $F_{RF}\left(w_{0},0,\phi \right)$ as a function of the azimuth angle, ϕ, measured in the yz-plane clockwise from the z-axis. The optical azimuth window function, cos(7ϕ), centered at ϕ=0 is also shown. The optical azimuth window function is much wider than the simulated $F_{RF}\left(w_{0},0,\phi \right)$, suggesting that the upconversion efficiency is mostly driven by the shape of the RF fields.

We estimate the performance of the receiver based on the results of the HFSS simulation of the RF field and on the set of parameters summarized in Table 1. In our simulations, the RF input power of 1 W was assumed, which is the power propagating from the far right end of the waveguide in Figure 4 towards the resonator. The peak field value was found to be $E_{0}\approx$ 1800 kV/m, therefore, $\alpha_{W}\approx 3.1\times 10^{-13}$ W(m/V)${}^{2}$. The azimuthal overlap integral in (12) is found by numerically multiplying and integrating the curves in the right panel of Figure 6 to be approximately 226 kV/m. This yields $g\approx 3.91\times 10^{9}$ 1/s and $g_{0}\approx 2200$ m/(V s). Using $\alpha_{W}$, $g_{0}$ and the parameters from Table 1, we find that the shot noise contribution into (25) equals $0.19\times k_{B}T_{rec}$. From (18), we also find the photonic gain G≈6.3, and thus the thermal noise contribution equals $0.04\times k_{B}T_{rec}$, assuming the receiver temperature is near 300 K. Therefore, the overall receiver sensitivity is limited at $0.23\times k_{B}T_{rec}$.

To compare the approach underlying the proposed receiver with the state of the art experimental demonstrations, we need to use a figure of merit independent of the assumed input microwave and optical pump powers. One such figure of merit is the up-conversion photon-number efficiency normalized to the optical pump power:

(26) $$\eta_{N}=\frac{1}{P_{0}}\frac{\hbar \omega_{RF}}{P_{RF\;in}}\frac{P_{s}}{\hbar \omega }.$$

Using Equation (15) and values from Table 1, we find that our projected $\eta_{N}\approx 0.019$ mW${}^{-1}$ is about seven times higher than the experimental result reported in [16], or about four times higher than the experimental result reported in [20].

## 6. Discussion and Conclusions

We carried out a theoretical analysis and numeric modeling of a photonic receiver which can serve as a front end for a W-band (94 GHz) radar. This receiver performs a low-noise, coherent, frequency-resolving up-conversion of the returned radar signal from the W-band to the near-infrared optical signal. Detecting the optical signal instead of the RF signal brings about a great practical boon that leads to reducing both the radar’s noise and its SWaP.

The underlying physical concept of this photonic receiver—using a high-finesse whispering gallery resonator in the electro-optical modulator modality—is not in itself new. As discussed in the Section 1, various groups proposed various versions of this type of receiver for a wide range of RF, millimeter-wave, and Terahertz signals. The main motivation for that research had been the theoretically predicted possibility of greatly improving the detector sensitivity, even to the level of detecting individual microwave photons. This sensitivity can be quantified in terms of the noise temperature. For the ultra-sensitive receiver, this temperature is predicted to be significantly lower than the ambient temperature. However, so far, even reaching the ambient temperature has remained an elusive experimental goal.

In this paper, we predict reaching the noise temperature at the level of 0.35 of the ambient temperature, on the absolute temperature scale. The prediction is made based on conservative assumptions and does not require improving any receiver elements beyond the state of the art. Taking the ambient temperature to be 300 K, we obtain the receiver noise temperature of 69 K without using any cooling implements. Comparing this to the typical noise temperature of a W-band low-noise amplifier, which is optimistically around 600 K, we arrive at a factor of 8.7 in the sensitivity improvement our technology offers relative to the modern W-band radars. This improvement already comes with a significant SWaP reduction due to a different SWaP budget of the photonic elements compared to the microwave elements. Further SWaP reduction can be traded for some of the SNR, if desired. This combination of factors makes our receiver an appealing solution for applications on SmallSats and CubeSats platforms. We plan to continue this effort towards such applications and carry out experimental testing of the receiver prototype. 

## Figures and Tables

**Table 1:** The list of parameters used in the numeric estimates of receiver performance and predicted receiver characteristics.

Parameter	Symbol	Value	Units
Optical wavelength	λ	1558.6	nm
Resonator radius	R	490	μm
Rim radius	r	104	μm
Ordinary refractive index	$n_{o}$	2.1189	
Extraordinary refractive index	$n_{e}$	2.1231	
Electro-optic coefficient	$r_{51}$	20	pm/V
TM coupling rate	$\gamma_{TM}$	$2\times 10^{7}$	rad/s
TE coupling rate	$\gamma_{TE}$	$4\times 10^{8}$	rad/s
Pump power	$P_{0}$	10	mW
LO power	$P_{LO}$	2	mW
RF impedance	ρ	50	Ω
Photodiode responsivity	R	0.9	A/W
Differential mode number	Δm	7	
**Predicted Performance**	**Symbol**	**Value**	**Units**
Peak field value	$E_{0}$	1800	kV/m
RF coefficient	$\alpha_{W}$	$3.1\times 10^{-13}$	W(m/V)${}^{2}$
Coupling rate	*g*	$3.91\times 10^{9}$	1/s
Normalized coupling rate	$g_{0}$	2200	m/(V s)
Photonic gain	*G*	6.3	
Photon-number conversion efficiency	$\eta_{N}$	0.019	1/mW
Shot noise contribution		$0.19\times k_{B}T_{rec}$	W/Hz
Thermal noise contribution		$0.04\times k_{B}T_{rec}$	W/Hz
Receiver Sensitivity	$P_{RF_{min}}/\Delta F$	$0.23\times k_{B}T_{rec}$	W/Hz

## Abbreviations

The following abbreviations are used in this manuscript:

SWaP	Size Weight and Power
TRMM	Tropical Rainfall Measuring Mission
GPM	Globabl Precipitation Measurement
WGM	Whispering Gallery Mode
RF	Radio Frequency
TE	Transverse Electric
TM	Transverse Magnetic
HFSS	High Frequency Structure Solver
PEC	Perfect Electric Conductor
FSR	Free Spectral Range
SNR	Signal to Noise Ratio
LO	Local Oscillator
